# Association of Postoperative Clinical Outcomes With Sarcopenia, Frailty, and Nutritional Status in Older Patients With Colorectal Cancer: Protocol for a Prospective Cohort Study

**DOI:** 10.2196/16846

**Published:** 2021-08-17

**Authors:** Nia Angharad Humphry, Thomas Wilson, Michael Christian Cox, Ben Carter, Marco Arkesteijn, Nicola Laura Reeves, Scott Brakenridge, Kathryn McCarthy, John Bunni, John Draper, Jonathan Hewitt

**Affiliations:** 1 School of Medicine Cardiff University Cardiff United Kingdom; 2 Institute of Biological, Environmental & Rural Sciences Aberystwyth University Aberystwyth United Kingdom; 3 Department of Surgery College of Medicine University of Florida Gainesville, FL United States; 4 Department of Biostatistics and Health Informatics Institute of Psychiatry, Psychology and Neuroscience King's College London United Kingdom; 5 Department of Surgery Cardiff and Vale University Health Board Cardiff United Kingdom; 6 Department of Surgery Harborview Medical Center University of Washington Seattle, WA United States; 7 North Bristol National Health Service Trust Bristol United Kingdom; 8 Royal United Hospitals Bath National Health Service Foundation Trust Bath United Kingdom; 9 Division of Population Medicine Cardiff University Cardiff United Kingdom

**Keywords:** sarcopenia, frailty, nutritional status, urine metabolomics, surgery, geriatric medicine

## Abstract

**Background:**

Older patients account for a significant proportion of patients undergoing colorectal cancer surgery and are vulnerable to a number of preoperative risk factors that are not often present in younger patients. Further, three preoperative risk factors that are more prevalent in older adults include frailty, sarcopenia, and malnutrition. Although each of these has been studied in isolation, there is little information on the interplay between them in older surgical patients. A particular area of increasing interest is the use of urine metabolomics for the objective evaluation of dietary profiles and malnutrition.

**Objective:**

Herein, we describe the design, cohort, and standard operating procedures of a planned prospective study of older surgical patients undergoing colorectal cancer resection across multiple institutions in the United Kingdom. The objectives are to determine the association between clinical outcomes and frailty, nutritional status, and sarcopenia.

**Methods:**

The procedures will include serial frailty evaluations (Clinical Frailty Scale and Groningen Frailty Indicator), functional assessments (hand grip strength and 4-meter walk test), muscle mass evaluations via computerized tomography morphometric analysis, and the evaluation of nutritional status via the analysis of urinary dietary biomarkers. The primary feasibility outcome is the estimation of the incidence rate of postoperative complications, and the primary clinical outcome is the association between the presence of postoperative complications and frailty, sarcopenia, and nutritional status. The secondary outcome measures are the length of hospital stay, 30-day hospital readmission rate, and mortality rate at days 30 and 90.

**Results:**

Our study was approved by the National Health Service Research Ethics Committee (reference number: 19/WA/0190) via the Integrated Research Application System (project ID: 231694) prior to subject recruitment. Cardiff University is acting as the study sponsor. Our study is financially supported through an external, peer-reviewed grant from the British Geriatrics Society and internal funding resources from Cardiff University. The results will be disseminated through peer-review publications, social media, and conference proceedings.

**Conclusions:**

As frailty, sarcopenia, and malnutrition are all areas of common derangement in the older surgical population, prospectively studying these risk factors in concert will allow for the analysis of their interplay as well as the development of predictive models for those at risk of commonly tracked surgical complications and outcomes.

**International Registered Report Identifier (IRRID):**

PRR1-10.2196/16846

## Introduction

### Background

There are numerous preoperative factors that affect surgical patients. In particular, older patients undergoing surgery have age-related factors that can affect their surgical outcomes. This is particularly important in the context of colorectal cancers, which are the third most diagnosed malignancy and the fourth leading cause of cancer death worldwide [1]. The majority of colorectal cancers are still diagnosed after the age of 65 years [2]. As populations continue to age, the incidence of major colon and rectal resections for cancer in the older population are projected to increase dramatically [3]. This makes it important to understand surgical risk factors in older adults and implement timely intervention where possible. Further, three of the most important age-related preoperative factors in older surgical patients are frailty, sarcopenia, and malnutrition. Although each has been studied individually, there is little information on their associations in older surgical patients.

### Frailty

At present, while there are no universally agreed upon consensus criteria for defining frailty, it can be thought of as physiologic decline and an increased risk of poor health resulting from aging [4]. There are numerous definitions, which vary from clinical phenotypes with specific required criteria to operational definitions (eg, the tally of an accumulation of deficits in older patients) [5,6].

Regardless of the definition used, preoperative frailty has been shown to be associated with poor postoperative outcomes in major colorectal surgery, such as an increased number of postoperative complications, an increased length of hospital stay, higher readmission rates, and decreased long-term survival rates [7]. Frailty is also associated with an increased cost of elective surgical care [8].

Two simple-to-use frailty assessments are the Clinical Frailty Scale (CFS) and the Groningen Frailty Indicator (GFI). Each of these assessments can be rapidly performed and have been validated with multiple cohorts [9,10].

### Sarcopenia

Sarcopenia is the age-related loss of muscle mass and function [11]. It is often associated with physical frailty, yet being clinically frail is not always a prerequisite for a sarcopenia diagnosis. It is also associated with malnutrition, although the two entities can occur separately [12].

Muscle function is often measured through hand grip strength, while lower extremity and torso muscle mass can be evaluated through numerous mechanisms, including computerized tomography (CT) morphometric analysis [13,14].

Sarcopenia has been associated with an increased risk of postoperative complications in patients undergoing gastrointestinal tumor resection, and it has a potential role in preoperative risk stratification if both muscle mass and function are assessed [15].

### Malnutrition

Malnutrition refers to deficiencies, imbalances, or excesses in a person’s intake of energy and nutrients [16]. In patients undergoing major abdominal surgery, malnutrition is associated with worse outcomes, including increased lengths of stay, increased in-hospital mortality rates, and higher costs of care [17]. The identification of malnutrition often relies on clinical screening tools that are reliant on various amounts of subjective recall [18]. A promising new strategy for making an objective diagnosis of dietary patterns that indicate a risk for malnutrition is urine metabolomics analysis [19]. This may play a role in assisting clinicians with identifying patients who may benefit from perioperative nutritional support.

### Study Overview

The objective of our feasibility study is to prospectively study older patients undergoing elective colorectal cancer resection and to provide informative data for further large-scale intervention studies. This study will evaluate the risk of postoperative complications (primary outcome), length of hospital stay (secondary outcome), and mortality (secondary outcome), with the following risk factors: frailty, sarcopenia and nutritional status.

The data obtained from this study may allow for the development of novel management strategies and targeted therapies of older surgical patients who experience a combination of frailty, sarcopenia, and malnutrition.

## Methods

### Setting

The Older Persons Surgical Outcomes Collaboration is a collaboration of surgeons, geriatricians, and epidemiologists who collect data on surgical outcomes in older individuals through multicenter research studies [20]. This collaborative collects data across the United Kingdom (sites include Cardiff, Bristol, Bath, Glasgow, Manchester, London, and Aberdeen) from all phases of surgical care, including longitudinal follow-ups. This study will initially enroll patients at three sites—Cardiff, Bristol, and Bath. It will then be registered on the Health & Care Research Wales Clinical Portfolio in order to allow other sites to enroll their patients. Cardiff University is acting as the study sponsor.

### Study Design

Our study is a prospective, multicenter, UK cohort study of older (aged ≥65 years) patients with colorectal cancer undergoing surgical resection. The objectives are to determine the association between clinical outcomes and frailty, nutritional status, and sarcopenia.

The inclusion criteria include patients with a diagnosis of colorectal cancer, those who plan to undergo surgical treatment for colorectal cancer, those aged ≥65 years, those with an abdominal CT scan that was taken prior to surgery (current standard of care), and those with the ability to understand the participant information sheet and are therefore able to provide written informed consent. Patients will be excluded if their procedural treatment is not performed with curative intent (including palliative colorectal stent insertion or the treatment of locally advanced tumors not amenable to curative resection) or if they are participating in another research study.

### Subject Recruitment

Patients will be identified by the usual clinical team as part of the routine, preoperative, multidisciplinary team meeting (the cancer multidisciplinary team). After patients are identified by screening, they will be approached by the colorectal clinical nurse specialist (CNS) by phone to discuss the study. If a patient agrees to discuss the study, the CNS will send the patient a participant information sheet via postal mail to allow for time for reviews and the further consideration of participation prior to the patient attending their preoperative assessment clinic (POAC) appointment. As the POAC is a routine standard-of-care appointment for evaluating a patient’s appropriateness for undergoing anesthesia and surgery, it does not subject patients to unnecessary visits. The CNS will also inform the study team about patient interest, so that a member of the study team can attend the POAC to answer patients’ questions and obtain written informed consent.

### Sample Size Justification

Based on an a priori power analysis, we will estimate the true postoperative incidence of complications (estimated to be 20%) with a 95% CI of ±11%. To achieve this, 50 patients will be enrolled. Due to the nature of the study, we anticipate that all patients will be followed up.

Enrollment will occur over a 12-month period beginning in winter 2020.

### Data Procurement and Management

Routine clinical data will be prospectively collected, including baseline demographics (age, sex, and race), height, weight, BMI, and medical comorbidities. Standard-of-care POAC laboratory results will be obtained, including full blood counts, basic metabolic panels, liver function test results, and C-reactive protein levels. Data on study specific characteristics, including urinary biomarkers for the assessment of dietary intake, radiographic muscle evaluations, and frailty assessments, will be recorded. At 90 days following surgery, the following specific outcomes will be reviewed: the length of hospital stay, readmissions to the hospital within 30 days, postoperative complications (evaluated using the Clavien-Dindo classification system), 30-day mortality, and 90-day mortality [21].

Each research site will maintain separate databases, and electronic records will be stored via standard National Health Service encryption. If hard copy data exist, they will be stored securely by each participating site’s principal investigator. All data shared with the centralized coordinating center will be deidentified and shared through a secure electronic database. All identifiable participant data will be deidentified by assigning research numbers to participants. Clinical data (including images) will be anonymized and securely stored centrally at Cardiff University for long-term storage in accordance with local guidelines (stored for 15 years). Consent forms will be stored centrally at Cardiff University.

### Outcomes

#### Coprimary Outcomes

The primary feasibility outcome is the estimation of the incidence rate of postoperative complications. The primary clinical outcome is the association between the presence of postoperative complications and frailty, sarcopenia, and nutritional status.

#### Secondary Outcomes

The secondary outcomes include the length of hospital stay, hospital readmission at 30 days, mortality at day 30, and mortality at day 90.

### Measuring Outcomes

#### Primary Outcome (Postoperative Complications)

Postoperative complications will be recorded and graded using the Clavien-Dindo classification system, categorized as grade 1 or grade 2 complications, and compared to grade 3 or higher complications [21].

#### Secondary Outcomes

Standard surgical and clinical outcomes (the length of stay, readmission to the hospital, and returns to theatre) will be tracked and recorded 90 days after surgery through a review of medical records.

### Measuring the Predictors

#### CT Morphometric Analysis

It is standard care for patients with colorectal cancer undergoing curative surgical resection to undergo preoperative CT scans for the evaluation of metastatic disease [22]. CT morphometric software allows for body composition analysis, in which the accurate estimation of total body muscle mass by using a single CT scan slice is performed [13,23]. Preoperative low muscle mass has been shown to be a predictor of poor outcomes for numerous surgical populations [15,24,25].

In order to identify radiographic evidence of sarcopenia, we will perform the CT morphometric assessment of the psoas and abdominal wall skeletal muscles in order to calculate the skeletal muscle index and psoas muscle index. This will be done by using SliceOmatic software (version 5.0, revision 7; TomoVision).

To calculate the total skeletal muscle cross-sectional area (cm^2^), all axial skeletal muscles (the psoas, paraspinal, and abdominal wall muscles) of a single CT slice at the third lumbar vertebra (where both transverse processes are visible) will be identified by using established Hounsfield unit attenuation thresholds (−19 to 150 Hounsfield units) for skeletal muscle. A skeletal muscle index (cm^2^/m^2^) will then be calculated by normalizing the total skeletal muscle cross-sectional area to the squared height of the patient. This same technique can be used to calculate a psoas muscle index (cm^2^/m^2^).

#### Urine Metabolomics Analysis

As previously stated, urine metabolomics provides clinicians with the ability to objectively measure dietary intake over time. Urine metabolomics profiles provide more objective results than dietary logs and questionnaires. Urine metabolomics profiles have been shown to vary among patients with controlled feeding conditions and can be used to classify the dietary intake of free-living individuals [26]. It has also been shown that urine metabolic profiles of individuals at home who are not undergoing strict dietary control can be quantified [27]. Multiple spot urine collections, such as the collection of a first-morning void, can provide metabolic profiles that are similar to those of 24-hour and temporally phased cumulative collections, thereby allowing for less rigorous requirements during home participation [28].

Urine samples will be collected over 4 separate, week-long time points; 3 samples will be collected during each time point, resulting in a total of 12 samples. The four time points consist of the week following the POAC visit, the immediate postoperative week, postoperative week 4, and postoperative week 8.

Participants will be provided with urine collection kits and prepaid, preaddressed envelopes so that they can return the samples by postal mail after each collection time point. If the participant is an inpatient for a given week, research staff will assist with sample collection. Patients will be phoned by the study team to remind patients about urine collections when they are outside of their hospital. If the patients are at home, each batch of the 3 samples will be stored in a domestic fridge until the end of the collection week.

Urine metabolomics analysis will be performed based on previously published methods [29]. Urine samples will be corrected for intraindividual variance by using specific gravity adjustments, and individuals’ samples will be pooled together for each collection week. Following extraction, the nontargeted metabolomics fingerprints of samples will be generated via flow infusion electrospray high-resolution mass spectrometry. The quantification of dietary biomarkers for measuring habitual dietary exposure and nutritional status will be performed by using liquid chromatography-triple quadrupole mass spectrometry via both the reverse phase and hydrophilic interaction liquid chromatography methods. Approximately 60 biomarkers that correspond to the intake of dietary components that are commonly consumed within the United Kingdom will be measured [30].

### Frailty and Functional Assessments

Multiple frailty and functional assessments will be performed at the POAC to evaluate a patient’s baseline status. The following two frailty assessments will be performed: The Canadian Study of Health and Aging CFS assessment and the GFI assessment.

The CFS is a 7-point scale that is used to score patients (from 1 to 7) based on the symptom burden of comorbidities and patients’ dependence on others for activities of daily living [10]. Patients with a score of 1 to 4 will be considered as “nonfrail,” while those with CFS scores of between 5 and 7 will be considered as “frail.”

The GFI is a simple, 15-point questionnaire that can be easily completed by clinical staff [31]. These assessments will be repeated during patients’ 8-week postoperative appointments to evaluate changes in frailty over time.

In addition to frailty assessments, numerous adjunctive functional tests will be performed at the POAC appointment. Strength will be assessed by using a hand grip dynamometer with the patient’s dominant hand and averaging the best results of 3 trials [5]. Mobility will be evaluated with a 4-meter walk test (measured from a starting standing position, at a normal walking pace, and with any usual walking aids) [32]. As strength and mobility are key components of frailty and the loss of muscle function is a key component of sarcopenia, these two tests will provide adjunctive information to both our frailty assessments and measured muscle masses. These functional assessments will also be repeated at the 8-week postoperative appointment to evaluate changes over time.

Finally, as an adjunct to both frailty evaluations and urine metabolomics analyses, the Mini Nutritional Assessment–Short Form will be used at both the POAC and 8-week follow-up visits. The Mini Nutritional Assessment–Short Form is a quick screening tool that is used to identify older patients that are malnourished or at risk of malnutrition [33], which has been shown to be associated with frailty in hospitalized patients [34]. Its use in this study will allow for comparisons between this subjective malnutrition assessment and objective urine metabolomics profiles.

### Data Analysis

A baseline descriptive analysis will be carried out for all patients who consent to the study, and this will be used to summarize the extent of missing data [35].

#### Primary Outcome: Estimating the Incidence of Postoperative Complications

This primary outcome will be estimated by using an asymptotic method to summarize the incidence of postoperative complications and calculate the associated 95% CIs.

#### Primary Outcome: Associating Risk Predictors With Postoperative Complications

A crude logistic regression model will be used to estimate the odds ratios for postoperative complications and will be fitted by comparing risk factors (eg, frailty).

#### Secondary Outcomes

Dichotomous outcomes will be analyzed in a similar manner as the primary outcome. If fewer than 8 cases are observed, the analysis will be reverted to a Fisher exact test.

The length of hospital stay will be shown by using a Kaplan-Meier plot with a survival function and will be analyzed via a Cox proportional hazards regression. Patient deaths will be censored on the date of death.

#### Missing Data and Populations Under Investigation

Missing data will be summarized, and the reasons will be explained.

#### Subgroup Analyses

Whether descriptive analyses will be carried out for subgroups will be determined at the time of analysis.

#### Software

Stata, version 15 (or later; StataCorp LLC) will be used to conduct the statistical analysis.

## Results

Our study was approved by the National Health Service Research Ethics Committee (reference number: 19/WA/0190) via the Integrated Research Application System (project ID: 231694) prior to subject recruitment.

Cardiff University is acting as the study sponsor.

Our study is financially supported through an external, peer-reviewed grant from the British Geriatrics Society and internal funding resources from Cardiff University.

The results will be disseminated through peer-review publications, social media, and conference proceedings.

## Discussion

Older patients represent a unique subset of patients who require major oncological surgery, as they are predisposed to a number of preoperative risk factors that are not often seen in younger patients. The purpose of our study is to evaluate older patients undergoing elective colorectal cancer resection by focusing on the three aforementioned perioperative factors—frailty, sarcopenia, and nutritional status. Following a series of sequential objective measurements, associations between each risk factor as well as associations between risk factors and common postoperative outcomes of interest will be examined.

Our preliminary study will provide data on the feasibility of obtaining serial urine samples for the metabolomics analysis of nutritional status in the perioperative period. We also believe that the data obtained from our study (and subsequent larger studies) will enable clinicians to identify older surgical patients who are the most at risk for poor surgical outcomes. These data will help establish which preoperative risk factors would be most beneficial to target in the future and will help clinicians provide better care to older surgical patients through more personalized and bespoke medicine.

## Abbreviations

CFS: Clinical Frailty Scale
CNS: clinical nurse specialist
CT: computerized tomography
GFI: Groningen Frailty Index
POAC: preoperative assessment clinic
